# Preparation and Antibacterial Activity Evaluation of 18-*β*-glycyrrhetinic Acid Loaded PLGA Nanoparticles

**Published:** 2015

**Authors:** Behrad Darvishi, Saeed Manoochehri, Golnaz Kamalinia, Nasrin Samadi, Mohsen Amini, Seyyed Hossein Mostafavi, Shahab Maghazei, Fatemeh Atyabi, Rassoul Dinarvand

**Affiliations:** a *Department of Pharmaceutics, Faculty of Pharmacy, Tehran University of Medical Sciences, Tehran, Iran.*; b *Drug and Food Control Department, Faculty of Pharmacy, Tehran University of Medical Sciences, Tehran, Iran.*; c *Department of Medicinal Chemistry, Faculty of Pharmacy, Tehran University of Medical Sciences, Tehran, Iran.*; d *Nanotechnology Research Centre, Faculty of Pharmacy, Tehran University of Medical Sciences, Tehran, Iran.*

**Keywords:** PLGA, Nanoparticle, 18-*β*-glycyrrhetinic acid, Antibacterial activity, *P. aeuroginosa*, *S. aureus*; *S. epidermidis*

## Abstract

The aim of the present study was to formulate poly (lactide-co-glycolide) (PLGA) nanoparticles loaded with 18-*β*-glycyrrhetinic acid (GLA) with appropriate physicochemical properties and antimicrobial activity. GLA loaded PLGA nanoparticles were prepared with different drug to polymer ratios, acetone contents and sonication times and the antibacterial activity of the developed nanoparticles was examined against different gram-negative and gram-positive bacteria. The antibacterial effect was studied using serial dilution technique to determine the minimum inhibitory concentration of nanoparticles. Results demonstrated that physicochemical properties of nanoparticles were affected by the above mentioned parameters where nanoscale size particles ranging from 175 to 212 nm were achieved. The highest encapsulation efficiency (53.2 ± 2.4%) was obtained when the ratio of drug to polymer was 1:4. Zeta potential of the developed nanoparticles was fairly negative (-11±1.5). *In-vitro* release profile of nanoparticles showed two phases: an initial phase of burst release for 10 h followed by a slow release pattern up to the end. The antimicrobial results revealed that the nanoparticles were more effective than pure GLA against *P. aeuroginosa*, *S. aureus* and *S. epidermidis*. This improvement in antibacterial activity of GLA loaded nanoparticles when compared to pure GLA may be related to higher nanoparticles penetration into infected cells and a higher amount of GLA delivery in its site of action. Herein, it was shown that GLA loaded PLGA nanoparticles displayed appropriate physicochemical properties as well as an improved antimicrobial effect.

## Introduction

Discovery and development of methods for targeted delivery of antibiotics to infected cells and the foci of bacterial infections has received a great deal of attention during the recent years. This is especially important regarding the fact that intracellular infections are difficult to eradicate (1) and infected cells and tissues are considered as microorganisms reservoirs, which may release them from time to time resulting in the recurrence of various systemic infections (2). Various colloidal systems such as niosomes, liposomes, microemulsions and micro and nanoparticles are amongst the most important drug delivery systems which are utilized for the preparation of such targeted carriers (3, 4). For instance Lutwyche *et al.* achieved a higher intracellular antibiotic delivery by gentamicin encapsulation in a liposomal drug delivery system and an increased therapeutic activity against intracellular pathogens was achieved (5).

To efficiently eradicate pathogens, an antibiotic should be able to be systemically transported in a form that can be endocytosed by phagocytes and the antimicrobial agent should be released inside the cells (6). In this respect, nanoparticles have a great value as they are rapidly picked up by mononuclear phagocytic cells when administered intravenously. Mononuclear phagocytic cells are kept responsible for providing a sanctuary and shelter for the intracellular bacteria (7). Therefore antibiotic encapsulation inside the nanoparticles has been suggested as a potential strategy for the management of various intracellular infections (8).

Polymeric nanoparticles (NPs) have several advantages which make them distinctive among various nanoparticulate systems. These advantages include their structural stability, narrow size distribution and the possibility of their functionalization for targeted drug delivery (9). Various polymers may be utilized for polymeric NPs preparation, among which PLGA or poly (lactic-co-glycolic acid) has a special place. PLGA has received FDA approval as a biocompatible and also biodegradable polymer and is commonly used in pharmaceutical industry and research as a desirable drug carrier (10, 11). 

An advantage of PLGA copolymers is that their degradation rate varies from months to years. There have been several reports of antimicrobial agents loaded PLGA NPs in the literature indicating their improved pharmacological and pharmacokinetic behavior when compared to their untreated counterparts (10). PLGA NPs may also have the potential to be utilized for oral and parenteral administrations. For instance oral administration of PLGA loaded curcumin NPs increased drug bioavailability at different levels (12). In another study, rifampicin, isoniazid, pyrazinamide, and ethambutol encapsulated PLGA NPs where used orally against *Mycobacterium Tuberculosis* and results showed enhanced bioavailability and improved pharmacodynamics when compared to the related free drug counterparts (13).

Parenteral administration of PLGA NPs loaded phosphorothioate antisense oligonucleotide nanoparticles for HIV treatment was also found to be successful and protected oligonucleotides from degradation (13). 

Antimicrobial agents delivery to bacteria by NPs may be based on two different approaches. In the first approach, nanoparticles are fused with the bacterial cell wall or membrane and the carried antimicrobial agent is subsequently released across the cell wall or cell membrane. In the second approach, nanoparticles are bound to cell wall and serve as a drug depot which continuously releases the anti-microbial drug molecules. The released anti-microbial agent will diffuse into the interior compartments of the bacteria (13).

18-*β*-glycyrrhetinic acid (GLA) is a pentacyclictriterpenoid derivative of beta-amyrin which is present in highly pure extracts of rhizomes of *glycyrrhiza glabra*. Glycyrrhetinic acid has a range of pharmacological properties including calmative, anti-inflammatory and antiallergic effects as well as antitumor properties. Moreover antibacterial and antifungal activities have been reported for this naturally occurring substance. It is shown that GLA can hinder DNA replication and may result in an inhibition in microbial toxins and enzymes production (14).

It has been demonstrated that high acute exposure to GLA would cause brief systemic changes, including an increased level of potassium excretion along with an enhanced sodium and water retention. Furthermore, following the administration of GLA, various side effects such as weight gain, alkalosis, renin-angiotensin-aldosterone system suppression, hypertension and muscular paralysis may occur. By physical encapsulation of the drug molecules inside the nanoparticulate systems, pharmacokinetics and therapeutic index of drugs significantly improve. Furthermore, a lower *in-vivo* toxicity is achieved due to reduced accumulation of GLA in kidney and liver (15).

To our best knowledge, there are currently no studies available on the formulation of GLA containing polymeric nanoparticles with the intention of enhancing GLA antibacterial activity. The aim of the present study was to develop a PLGA based nanocarrier system for GLA to enhance the antibacterial effects of GLA and to minimize its side effects. The developed nanoparticles have the potential to target infected tissues and phagocytic cells and exert their antibacterial effect on various microorganisms. *Staphylococcus aureus* (*S. aureus*)*, Staphylococcus epidermidis *(*S. epidermidis*) and *Pseudomonas aeruginosa* (*P. aeruginosa*) were chosen as model bacteria for evaluating the effect of the developed nanoparticles on microbial organisms as they are considered as the most significant bacteria involved in various infectious diseases in human being and are internalized in phagocytic cells (-). *S. aureus* is a widespread pathogen which is considered to be responsible for food poisoning and nosocomial infections (21). *S. epidermidis* is another pathogen which is involved in extraneous devices and implants related infections due to its particular capability of growing biofilms on polymer surfaces (22). *P. aeruginosa* is a gram negative bacterium which is another common cause of nosocomial infections, where only a limited number of antibacterial agents show an acceptable defense against this organism (23). The antimicrobial activity of the nanoparticles was further compared with pure GLA against different gram-negative and gram-positive bacteria.

## Experimental

*Materials*

GLA powder was purchased from Sigma-Aldrich (St. Louis, MO, USA). PLGA (50:50 ratio lactide: glycolide, Resomer RG 504 H, MW: 48 kDa) was obtained from Boehringer Ingelheim (Ingelheim, Germany). Polyvinyl alcohol (PVA) was from Acros (Geel, Belgium). Mueller-Hinton broth and Mueller-Hinton agar media (both from Merck, Darmstadt, Germany) were used for microbiological tests. *S. aureus* (ATCC number: 29737), *P. aeroginosa* (ATCC number: 9027), *Escherichia coli (E.coli)* (ATCC number: 35218) and *S. epidermidis* (ATCC number: 12229) were acquired from the department of drug and food control (Faculty of Pharmacy, Tehran University of Medical Sciences, Tehran, Iran). All other materials used in this study were of analytical or HPLC grade.

*Preparation of GLA loaded PLGA nanoparticles*

PLGA NPs were fabricated using a sonication/ solvent evaporation method (9). In the first step, PVA (0.5% w/w) was dissolved in deionized water forming the aqueous phase and different ratios of GLA to PLGA were dissolved in acetone at room temperature (25 °C) To prepare organic phase (oil phase). The prepared oil phase was added drop wise to the aqueous phase while sonicating using an ultrasonic probe (Misonix, Farmingdale, NY, USA) equipped with a microtip (power: 11 watt). The entire system was located in an ice bath to avoid any loss of oil phase as a result of organic solvent evaporation during sonication. The above mixture was subsequently magnetically stirred overnight at room temperature where acetone was removed under constant stirring condition. Finally the solution was centrifuged three times (Sigma 3K30, Osterode, Germany) at 21000 rpm for 30 min in order to remove any extra PVA and was then lyophilized (Christ Alpha 1-4, Osterode, Germany). The final dry powder was used for physicochemical and antibacterial investigations.

*Nanoparticle size, morphology and zeta potential*

For measuring the size, polydispersity index and zeta potential of nanoparticles, lyophilized nanoparticles were dissolved in deionized water and were assessed by photon correlation spectroscopy (PCS) using a Zeta sizer nano ZS (Malvern Instruments, Worcestershire, UK) at 25 °C. All the experiments were accomplished in triplicate and Mean±SD was reported.

Surface morphology and shape of nanoparticles were investigated by scanning electron microscopy (SEM, Philips XL 30, Philips, the Netherlands). A small amount of freeze dried powder of NPs was fixed on a double-sided tape attached to a metallic sample stand, and was gold coated before SEM (9).

*Encapsulation efficiency*

5 mg lyophilized NPs were dispersed in 1 mL acetonitrile and were shaken for 30 seconds and were further sonicated for approximately 5 min. 2 mL methanol was then added to the mixture in order to precipitate the polymer. The sample was then centrifuged at 21000 rpm for 20 min and the free GLA containing supernatant was separated for further analysis. Drug quantity in the supernatant was determined by a UV spectrophotometry method. Drug loading was calculated as the GLA content in NPs divided by the total weight of NPs. The encapsulation efficiency was obtained by calculating the ratio of GLA amount incorporated inside the NPs to the GLA amount which was used for NPs preparation (9). Each experiment was repeated three times.

*Differential scanning calorimetry*

Differential scanning calorimetry (DSC) was performed for PLGA, GLA, their physical mixture and GLA loaded PLGA nanoparticles with a Mettler DSC 823 unit (Mettler Toledo, Greifensee, Switzerland) and a Julabo thermocryostate model FT100Y (Julabo labor technik, Seelbach, Germany) with Indium used for instrument calibration. 2 mg of samples were placed into DSC aluminum pans and sealed. Pure water with the same amount was used as the reference. Sample scanning was performed with a temperature range of 30 to 400 °C and with a rate of 5 °C min^–1^ under a 8 kPa nitrogen atmosphere (n=3) (9).

*Release evaluation*

Drug release evaluation was performed according to a previously published method for piroxicam nanoparticles (24) with some modifications. 5 mg of lyophilized GLA loaded PLGA NPs was dispersed in 10 mL phosphate buffer saline (PBS, pH=7.4) and placed in a dialysis tube (cut-off: 12 kDa), and was floated in a flask containing 50 mL Phosphate Buffered Saline (PBS) medium. The entire system was located at 37 °C in a beaker (250 rpm). After a predetermined period, the old medium was fully removed and fresh medium was replaced each time. 5 mL of the old medium was analyzed by UV spectrophotometry for its GLA content. The release profile of free GLA was also evaluated by a dialysis bag under the same conditions.

*Antibacterial activity of GLA loaded PLGA nanoparticle suspensions*

Minimum Inhibitory Concentration (MIC) or the lowest concentration of GLA which is able to inhibit microorganisms outgrowth was determined for GLA loaded NPs using broth micro dilution method. GLA NPs suspensions with equivalent concentration of GLA based on their encapsulation efficiency were used against *S. aureus*, *S. epidermidis *and *P. aeroginosa*. Furthermore drug free NPs were utilized to see if the carrier or other formulation components exert any antibacterial activity which may interfere with the results. It is noteworthy that positive and negative control groups were also considered in this MIC determination study, where positive control groups did not contain any anti-microbial agent and negative control groups were not inoculated with any bacteria. Briefly, the lyophilized bacteria were first suspended in sterile water for injection and were then cultured in Luria Bertuni agar mediums (Scharlau, Spain) and were incubated for 24 h at 37 °C. A single colony from the plates was then transferred into 4 mL fluid of Luria Bertuni agar medium and was incubated at 37 °C and 200 rpm in a shaking incubator for an overnight period. In the next step centrifugation at 3000 rpm for 15 min at 4 °C was used for cellular harvesting. The supernatant was finally removed and the cellular sediment was washed two times with Ringer solution and was dispersed in the same medium in a concentration range of about 10^5^ to 10^6^ CFU/mL to be exploited for broth dilution method (25).

For each sample and each bacterial strain, eight tubes were employed and autoclaved at 121 °C for 15 min. The samples were then serially diluted in the tubes and 1 mL of inoculums of tested bacterium (final concentration of 10^5^-10^6^ CFU/mL) was introduced into the tubes. After 24 h of incubation at 37 °C, the tubes were evaluated according to their bacterial growth related turbidity and MIC was determined as the minimum GLA concentration without any visible bacterial growth (6). 

## Results and Discussion

*Preparation and characterization of GLA loaded nanoparticles*

**Figure 1:**
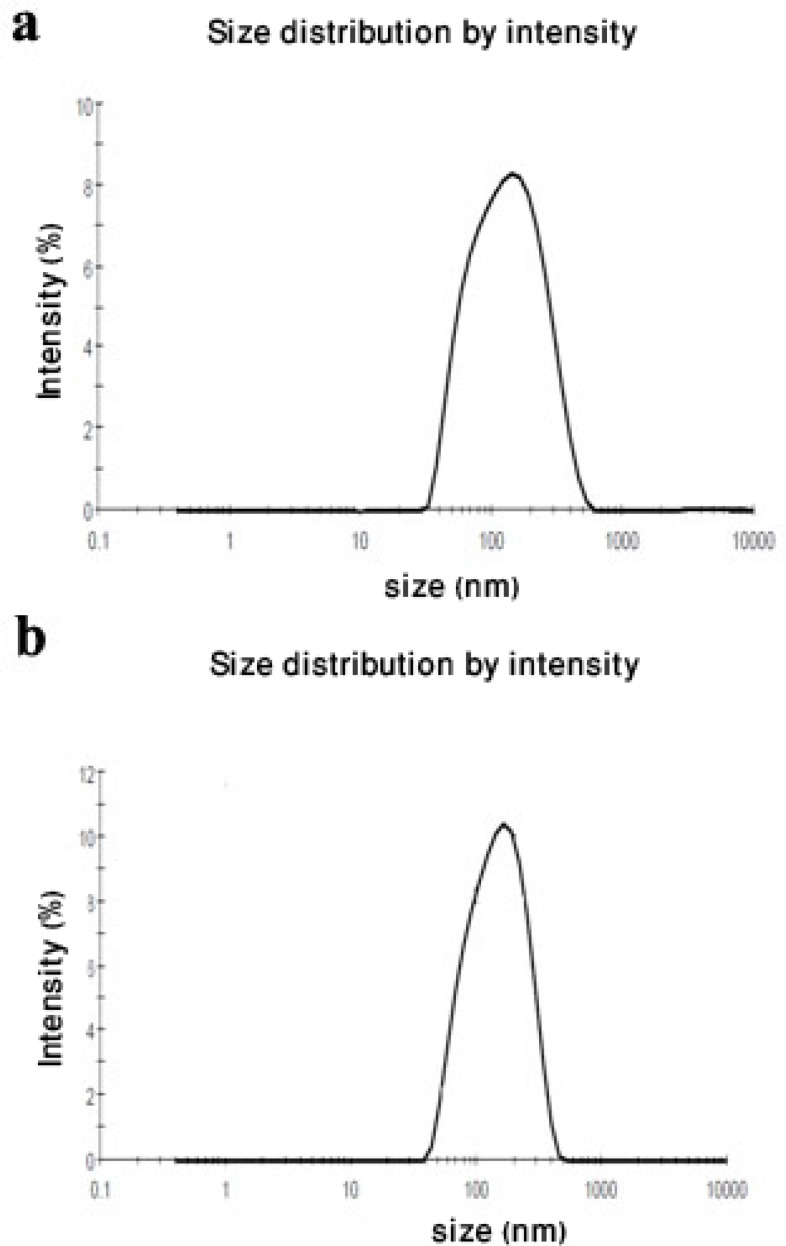
Particle size distribution of nanoparticles with drug to polymer ratios of [a] (1:1) and [b] (1:2) prepared by sonication/ solvent evaporation technique

GLA loaded NPs were prepared by sonication/ solvent evaporation method. Encapsulation efficiency, drug loading, size, polydispersity index and zeta potential of GLA loaded NPs are reported in Table1. According to Table 1, NPs size was in the range of 175 to 212 nm and the prepared nanoparticles were highly uniform and monodispersed (Figures 1a and 1b). By increasing the drug to polymer ratio from 1:1 to 1:4, size was increased from 184 to 212 nm. This result may be related to an increase in viscous forces of droplets which resists against droplets break down by sonication especially in higher polymer concentrations. These resisting forces stand against the shear forces in the oil phase and determine the final size and particle size distribution in nanoparticles (26). Furthermore, Table 1 shows that by increasing the amount of polymer, higher encapsulation efficiencies were achieved. Maximum encapsulation efficiency was attained when the amount of drug to polymer ratio was 1:4. As it is observed higher polymer concentration is related to a higher encapsulation efficiency which may be explained in two different ways. First, it can be proposed that when the polymer concentration is high, the polymer molecules may precipitate on the surface of the dispersed phase droplets. As a result drug molecules diffusion through the two phases boundary is restricted and a higher encapsulation efficiency is achieved (27). Alternatively, it may be proposed that a higher polymer concentration can increase the viscosity of the system and make a barrier toward the free diffusion of drug molecules through the boundary phase of polymeric droplets (28).

**Table 1:** PLGA nanoparticles characteristics (O: oil phase, W: water phase, PLGA: Poly (lactide-co-glycolide), PDI: polydispersity index and EE: encapsulation efficiency).

	**Drug: PLGA ratio (%)**	**O/W ratio (%)**	**Time of sonication** **(min)**	**Size** **(nm)**	**PDI**	**Zeta potential**	**EE**
1	1:1	10	5	184 ± 25	0.23 ± 0.02	-11.48 ± 1.8	28.3 ± 1.8
2	1:2	10	5	197 ± 19	0.15 ± 0.01	-13.71 ± 2.1	34.4 ± 2.1
3	1:3	10	5	204 ± 28	0.14 ± 0.01	-15.32 ± 2.3	44.7 ± 2.2
4	1:4	10	5	212 ± 29	0.20±0.02	-17.13 ± 1.9	53.2±2.4
5	1:1	5	5	207 ± 20	0.15 ± 0.02	-11.52 ± 1.5	29.3 ± 1.8
6	1:1	15	5	179 ± 22	0.17±0.01	-11.07 ± 1.8	28.4±1.7
7	1:1	20	5	176 ± 19	0.18 ± 0.02	-10.83 ± 1.3	28.9 ± 1.9
8	1:1	10	3	196 ± 22	0.16 ± 0.01	-11.08 ± 1.4	34.3 ± 2.1
9	1:1	10	7	180 ± 21	0.20 ± 0.02	-11.02 ± 1.7	24.7 ± 1.8
10	1:1	10	10	175 ± 15	0.14 ± 0.01	-11.53± 1.4	23.4 ± 1.6

Table 1 also shows the influence of the acetone content on the size of NPs. The ratio between continuous and dispersed phases may have an effect on the stability and size of nanoparticles. As shown in Table 1 by increasing the ratio of the oil phase, the size of nanoparticles is slightly decreased and their encapsulation efficiency and drug loading is slightly increased. It has been suggested that by increasing the ratio of aqueous phase, the external surface energy of oil droplets, will be dispersed in a higher volume and results in a lower droplet breakage and subsequently the particle size will be increased. It should be mentioned that GLA is poorly soluble in water and has a higher affinity for the oil phase in an oil in water emulsion which will lead to a higher entrapment efficiency and drug loading with increasing the oil phase ratio (9).

Another factor which was evaluated for its effects on the size of nanoparticles was sonication time which was between 1 and 20 min. The results shown in Table 1 suggest that an increase in the sonication time would result in a reduction in the size of nanoparticles. When a higher sonication time (10 min) is utilized, the high energy results in a rapid dispersion of oil droplets with a smaller size and a monomodal profile of distribution. The emulsification step is one of the most vital steps in the nanoparticles formation process. Inappropriate phase dispersion will result in the formation of larger particles with a wider particle size distribution. The NPs size is dependent on the size of droplets which are formed during the emulsification process. As a result by reducing the size of emulsion droplets, smaller nanoparticles can be achieved. 

Nanoparticles stabilization phase is a very significant step in the process of emulsification. Protection of nanodroplets is usually achieved by surfactant molecules utilization. Surfactant molecules prevent the occurrence of coalescence in nanodroplets and produce nanoparticles with a smaller particle size. Herein, a 0.5% concentration of PVA was used to stabilize the formed nanoparticles and develop some sort of coverage between the phases interface in oil in water emulsion.

Other than size, zeta potential is another major characteristic of nanoparticles which may have impacts on both nanoparticles stability and cellular adhesion. Zeta potential was also measured in PLGA NPs and as shown in Table 1, the values of negative zeta potential on the nanoparticles were reduced as the polymer concentration decreased from 1:4 to 1:1 drug to polymer ratio. This reduced negative charge may be attributed to the higher amount of unentrapped drug molecules with lower polymer concentrations and their shielding effect on the carboxylic moieties of PLGA molecules on the particle surface (29). It has been shown that the extent of phagocytosis is increased with an increase in NPs zeta potential where the minimum phagocytosis is related to the instance in which zeta potential is approximately zero (30). On the other hand, due to the negative surface charge of endothelial cells and their repulsive forces toward the negatively charged NPs, the half life of these NPs may increase in blood systemic circulation keeping the NPs more available to the phagocytic cells (31).

*Scanning electron microscopy*

**Figure 2:**
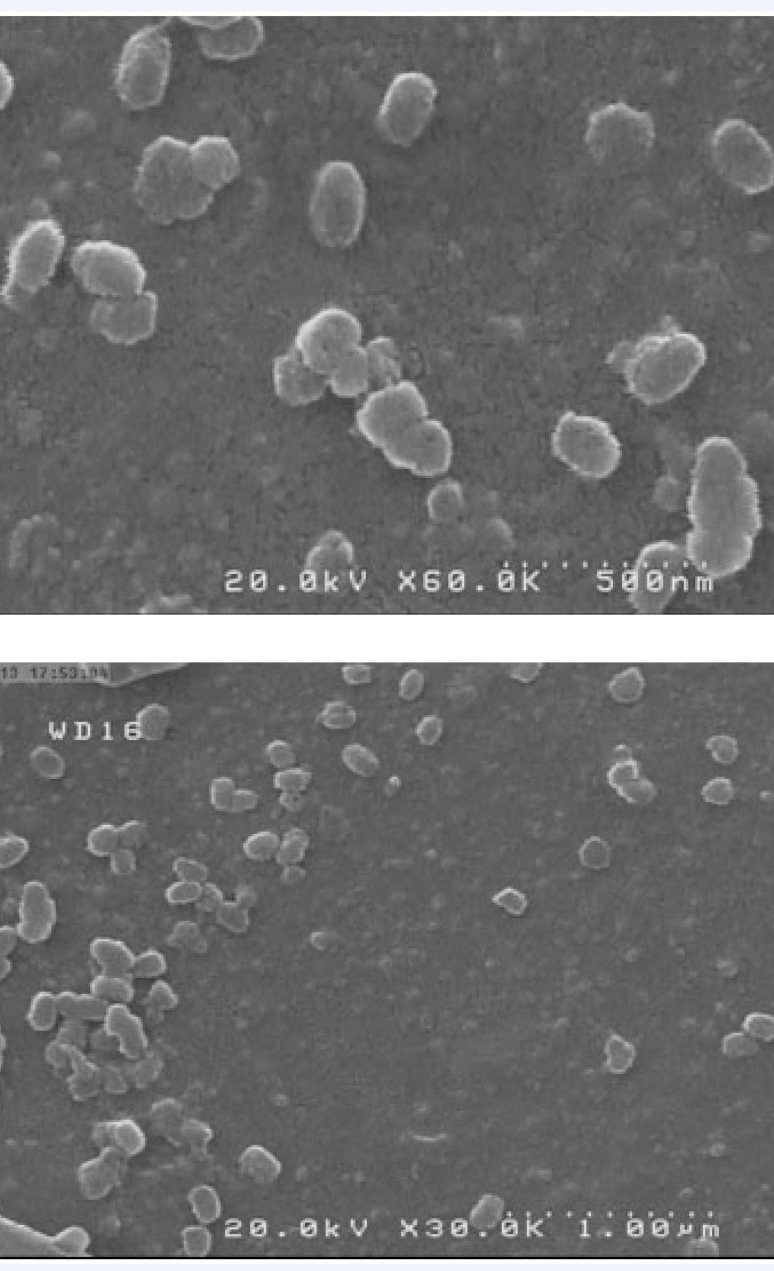
Scanning electron micrographs of 18-*β*-glycyrrhetinic acid (GLA) loaded poly (lactide-co-glycolide) (PLGA) nanoparticles with different magnifications.

SEM micrograph of GLA loaded NPs showed that NPs are roughly spherical and have smooth surfaces (Figure 2). All formulations appeared to be monodispersed and homogenous irrespective of their compositions. The NPs size obtained by photon correlation spectroscopy were larger than those observed by SEM which may be related to hydrodynamic diameter of swollen and inflated polymeric NPs in water (32).

*Differential scanning calorimetry*

**Figure 3:**
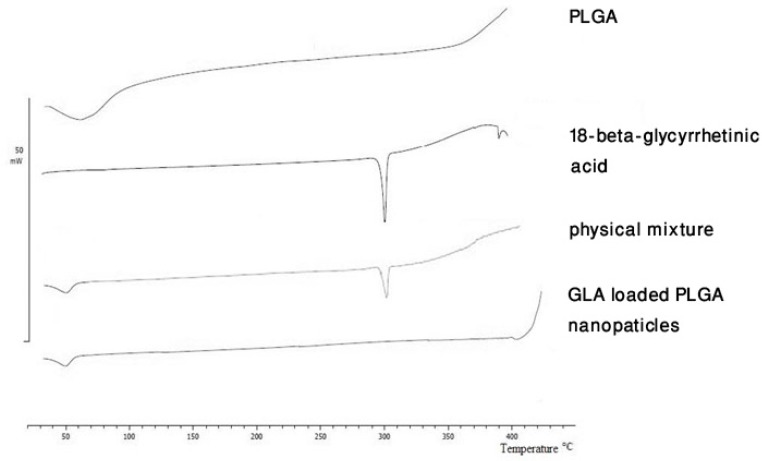
Differential scanning calorimetry thermograms of poly (lactide-co-glycolide) (PLGA), 18-*β*-glycyrrhetinic acid (GLA), their physical mixture and GLA loaded PLGA nanoparticles.

DSC was used to investigate the thermal behavior of formulations. Figure 3 shows that pure PLGA exhibits an endothermic peak at 60 °C which can be related to its relaxation peak following the glass transition phase. Due to the amorphous nature of PLGA, no melting point was observed in PLGA thermograms. The pure GLA demonstrated a sharp peak at 300 °C which can be related to its melting point. The DSC thermograms of the physical mixture of GLA and PLGA showed peaks which were the result of plain superposition of each of the two substances DSC thermograms. The enthalpy reduction for the physical mixture of drug and polymer can be explained by the presence of smaller amount of drug molecules in the mixture in comparison with pure drug. The DSC curve of GLA containing NPs did not show the endothermic peak of GLA which suggests that the drug is incorporated into the nanoparticles in a disordered and amorphous shape. Any severe alteration in the thermal behavior of either the polymer or the drug may be related to drug polymer interaction (33). In the current study no formation of any new peak and no shifting of any peak in the DSC thermogram was observed. 

*Release study*

**Figure 4:**
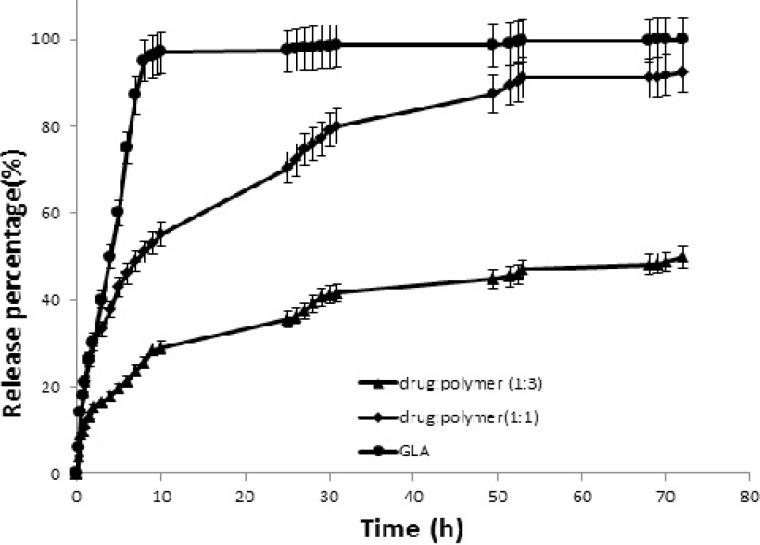
Release profile of free 18-*β*-glycyrrhetinic acid (GLA) and GLA loaded poly (lactide-co-glycolide) (PLGA) nanoparticles with different drug to polymer ratios.

The release profiles of pure GLA and GLA loaded NPs with different drug to polymer ratios are shown in Figure 4. *t*_30% _is the time needed for dissolving 30% of drug and is inversely related to the dissolution rate.* t*_30% _was 2 h and 9 h for NPs with 1:1 and 1:3 drug to polymer ratios, respectively. 

Figure 4 also demonstrates that GLA release from NPs was slower and more sustained than that of pure GLA. Drug release rate from various drug delivery systems is usually controlled by dissolution and/or diffusion (34). In this study the presence of water insoluble polymer in NPs matrix composition may interfere with the drug release from NPs where it can reduce the amount of water penetration in NPs and subsequently affect drug dissolution and diffusion. PLGA microparticles drug release is usually composed of two phases of initial burst release followed by a slower release phase. This burst release is of special importance as it affects microparticles toxicity and their therapeutic efficacy. The burst release is generally defined as the total amount of released drug from the particulate system through the first 24 h. this total amount of drug release is normally about 10 to 80% of the total drug loading in such particles (35, 36).

 In the present study, the release profiles of GLA from NP formulations followed a two phase pattern: in the first phase, an initial burst release occurs which takes about 4 h and is followed by the second slow release phase. The release curves demonstrated that the initial burst release level is dependent on the amount of PLGA polymer in NPs samples. The results showed that during the first 10 h, an initial burst release led to an early release of 100%, 55% and 29% of drug from pure GLA, GLA loaded NPs with 1:1 drug to polymer ratio and GLA loaded NPs with 1:3 drug to polymer ratio, respectively. The burst release of GLA may be related to the drug molecules that are poorly entrapped in the polymer matrix. It is noteworthy that similar observations were reported by other researchers working on paclitaxel and azithromycin PLGA NPs (37).

*Antibacterial activity of GLA nanoparticle suspensions*

**Figure 5:**
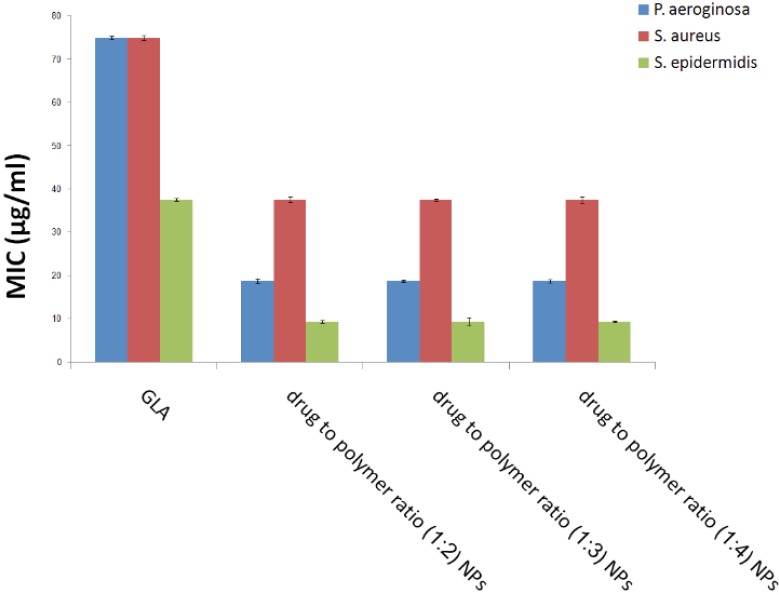
Minimal inhibitory concentration (MIC) of free 18-*β*-glycyrrhetinic acid (GLA) and GLA loaded poly (lactide-co-glycolide) (PLGA) nanoparticles with different drug to polymer ratios in three different bacterial strain

The MIC of GLA loaded PLGA NPs as well as free GLA was reported for *S. aureus*, *S. epidermidis* and *P. aeruginosa *(Figure 5). Drug free PLGA nanoparticles displayed no antibacterial effect which suggests that NPs components and ingredients did not have any considerable antibacterial activity. The MIC of GLA loaded NPs was approximately 2 times lower for *S. aureus*, 3 times lower for *S. epidermidis*, and 4 times lower for *P. aeroginosa* compared to free GLA solution regardless of drug to polymer ratio. This indicates that the effective dose of this antibiotic can be reduced through the administration of GLA loaded PLGA NPs against the above mentioned bacteria which will subsequently result in lower side effects.

Consequently, *in-vitro* antimicrobial activity of GLA loaded PLGA NPs was better than the free drug. It is noteworthy that an enhanced antimicrobial activity with antibiotics loaded PLGA NPs has been previously reported (6). This better antibacterial activity may be attributed to the improved penetration of NPs from biological membranes and its better access to the bacteria internalized within the phagocytes.

It has been revealed that phagocytic cells can endocytose NPs and can release the drug inside these cells (6). The GLA loaded NPs could be useful in drug targeting to phagocytic cells and can improve the treatment and management of intracellular infections compared to free antibiotics. Other kinds of NPs have been successfully evaluated for carious other routes of administration including ocular (38) and oral (39), and the formulated GLA loaded PLGA NPs may have the potential to be used for these routes of administration as well.

## Conclusion

GLA loaded PLGA nanoparticles were successfully prepared with an appropriate size and zeta potential. The antimicrobial activity evaluation showed that NPs were more effective than pure GLA against *S. aureus, S. epidermidis *and* P. aeroginosa*. It can be proposed that the clinical activity of GLA can be enhanced if a GLA nanoparticle formulation is used and this allows for a more efficient therapy compared to the conventional formulation of free antibiotics present in the market.
